# Supplementary material for: Dual recognition of structurally unrelated mildew effectors underlies the broad-spectrum resistance of Pm3e in wheat
Source: Nat Commun. 2026 May 2;17:5955. doi: 10.1038/s41467-026-72199-w (PMC13342611; doi:10.1038/s41467-026-72199-w)
Supplement: Supplementary file 2 — Descriptions of Additional Supplementary Files [file 41467_2026_72199_MOESM2_ESM.pdf]

Description of Additional Supplementary Files

Supplementary Dataset 1 Overview about isolates used in this study.

Supplementary Dataset 2 Summary of effector constructs used in this study.
Supplementary Dataset 3 Overview of predicted effector structures used in this study.

Supplementary Dataset 4 Details on statistical tests and exact p-values for all analysis in this study.
