# Supplementary material for: Dual recognition of structurally unrelated mildew effectors underlies the broad-spectrum resistance of Pm3e in wheat
Source: Nat Commun. 2026 May 2;17:5955. doi: 10.1038/s41467-026-72199-w (PMC13342611; doi:10.1038/s41467-026-72199-w)
Supplement: Supplementary file 7 — Reporting Summary [file 41467_2026_72199_MOESM7_ESM.pdf]

nature portfolio

Corresponding author(s): Lukas Kunz, Marion Müller, Beat Keller

Last updated by author(s): 24/2/26

# Reporting Summary

Nature Portfolio wishes to improve the reproducibility of the work that we publish. This form provides structure for consistency and transparency in reporting. For further information on Nature Portfolio policies, see our Editorial Policies and the Editorial Policy Checklist.

## Statistics

For all statistical analyses, confirm that the following items are present in the figure legend, table legend, main text, or Methods section.

| n/a | Confirmed | |
|---|---|---|
| ☐ | ☒ | The exact sample size (*n*) for each experimental group/condition, given as a discrete number and unit of measurement |
| ☐ | ☒ | A statement on whether measurements were taken from distinct samples or whether the same sample was measured repeatedly |
| ☐ | ☒ | The statistical test(s) used AND whether they are one- or two-sided<br>*Only common tests should be described solely by name; describe more complex techniques in the Methods section.* |
| ☒ | ☐ | A description of all covariates tested |
| ☐ | ☒ | A description of any assumptions or corrections, such as tests of normality and adjustment for multiple comparisons |
| ☐ | ☒ | A full description of the statistical parameters including central tendency (e.g. means) or other basic estimates (e.g. regression coefficient) AND variation (e.g. standard deviation) or associated estimates of uncertainty (e.g. confidence intervals) |
| ☐ | ☒ | For null hypothesis testing, the test statistic (e.g. *F*, *t*, *r*) with confidence intervals, effect sizes, degrees of freedom and *P* value noted<br>*Give P values as exact values whenever suitable.* |
| ☒ | ☐ | For Bayesian analysis, information on the choice of priors and Markov chain Monte Carlo settings |
| ☒ | ☐ | For hierarchical and complex designs, identification of the appropriate level for tests and full reporting of outcomes |
| ☒ | ☐ | Estimates of effect sizes (e.g. Cohen's *d*, Pearson's *r*), indicating how they were calculated |

*Our web collection on statistics for biologists contains articles on many of the points above.*

## Software and code

Policy information about availability of computer code

| | |
|---|---|
| Data collection | Software used: Fusion Software (v17.02, Vilber Lourmat Fusion FX) |
| Data analysis | Microsoft Excel, bowtie2 (v2.4.4), FreeBayes (v1.3.6), blastn (v2.12.0), TM-align (v20220412), AlphaFold2, ColabFold_AlphaFold2, HHblits Toolkit, AlphaFold3, Foldseek ChimeraX (v1.8), InterPro, SignalP 4.0, SignalP 4.1, SignalP 5.0, SignalP 6.0, DeepTMHMM, Salmon (v1.4.0), edgeR (v3.40.2), IGV (v2.8.6), , Fiji/ImageJ (2.14.0/1.54p), R (4.4.3), RStudio (2024.12.1).<br>Python script for SNP calling: https://github.com/MarionCMueller/AD-assay/blob/main/scripts/Script_BSA_SNPcall.v8.py<br>R functions for G-test and BSA functions:https://github.com/MarionCMueller/AD-assay/blob/main/scripts/BSA_Blumeria_functions.R |

For manuscripts utilizing custom algorithms or software that are central to the research but not yet described in published literature, software must be made available to editors and reviewers. We strongly encourage code deposition in a community repository (e.g. GitHub). See the Nature Portfolio guidelines for submitting code & software for further information.

## Data

Policy information about availability of data

All manuscripts must include a data availability statement. This statement should provide the following information, where applicable:

- Accession codes, unique identifiers, or web links for publicly available datasets
- A description of any restrictions on data availability
- For clinical datasets or third party data, please ensure that the statement adheres to our policy

Sequencing reads produced in this study for the avirulence depletion assay are deposited at the sequence read archive under accession number PRJNA1125842.

Sequencing reads of the mutant Bgt_FAB are available under accession number PRJNA1016363. Predicted protein structures of Bgt effectors are available at Zenodo (https://doi.org/10.5281/zenodo.17276912). Source data are provided with this paper.

## Research involving human participants, their data, or biological material

Policy information about studies with human participants or human data. See also policy information about sex, gender (identity/presentation), and sexual orientation and race, ethnicity and racism.

| | |
|---|---|
| Reporting on sex and gender | *Use the terms sex (biological attribute) and gender (shaped by social and cultural circumstances) carefully in order to avoid confusing both terms. Indicate if findings apply to only one sex or gender; describe whether sex and gender were considered in study design; whether sex and/or gender was determined based on self-reporting or assigned and methods used. Provide in the source data disaggregated sex and gender data, where this information has been collected, and if consent has been obtained for sharing of individual-level data; provide overall numbers in this Reporting Summary. Please state if this information has not been collected. Report sex- and gender-based analyses where performed, justify reasons for lack of sex- and gender-based analysis.* |
| Reporting on race, ethnicity, or other socially relevant groupings | *Please specify the socially constructed or socially relevant categorization variable(s) used in your manuscript and explain why they were used. Please note that such variables should not be used as proxies for other socially constructed/relevant variables (for example, race or ethnicity should not be used as a proxy for socioeconomic status). Provide clear definitions of the relevant terms used, how they were provided (by the participants/respondents, the researchers, or third parties), and the method(s) used to classify people into the different categories (e.g. self-report, census or administrative data, social media data, etc.) Please provide details about how you controlled for confounding variables in your analyses.* |
| Population characteristics | *Describe the covariate-relevant population characteristics of the human research participants (e.g. age, genotypic information, past and current diagnosis and treatment categories). If you filled out the behavioural & social sciences study design questions and have nothing to add here, write "See above."* |
| Recruitment | *Describe how participants were recruited. Outline any potential self-selection bias or other biases that may be present and how these are likely to impact results.* |
| Ethics oversight | *Identify the organization(s) that approved the study protocol.* |

Note that full information on the approval of the study protocol must also be provided in the manuscript.

# Field-specific reporting

Please select the one below that is the best fit for your research. If you are not sure, read the appropriate sections before making your selection.

☒ Life sciences ☐ Behavioural & social sciences ☐ Ecological, evolutionary & environmental sciences

For a reference copy of the document with all sections, see nature.com/documents/nr-reporting-summary-flat.pdf

# Life sciences study design

All studies must disclose on these points even when the disclosure is negative.

| | |
|---|---|
| Sample size | The sample size of each experiment is listed in the figure legend and or the method section. |
| Data exclusions | For detached leaf phenotyping experiments, individual leaf segments were excluded from scoring if they were overgrown with mold fungi, as this prevented accurate disease assessment. The robustness of the phenotyping was ensured by evaluating at least three biological replicates per genotype. For the Nicotiana benthamiana experiments and the western blotting analyses, no data were excluded. For the particle bombardment assays, experimental replicates in which not all treatment groups contained at least 25 interactions were excluded, as sample sizes below this threshold were considered insufficient for drawing robust conclusions. |
| Replication | All hypersensitive response assessment experiments in Nicotiana benthamiana and all western blotting experiments were performed three times. For the Bgt phenotyping experiments on detached leaf assays, at least three independent biological replicates were scored. |
| Randomization | No randomization was performed. |
| Blinding | No blinding was performed. |

# Reporting for specific materials, systems and methods

We require information from authors about some types of materials, experimental systems and methods used in many studies. Here, indicate whether each material, system or method listed is relevant to your study. If you are not sure if a list item applies to your research, read the appropriate section before selecting a response.

## Materials & experimental systems

| n/a | Involved in the study |
|---|---|
| | ☒ Antibodies |
| ☒ | ☐ Eukaryotic cell lines |
| ☒ | ☐ Palaeontology and archaeology |
| ☒ | ☐ Animals and other organisms |
| ☒ | ☐ Clinical data |
| ☒ | ☐ Dual use research of concern |
| | ☒ Plants |

## Methods

| n/a | Involved in the study |
|---|---|
| ☒ | ☐ ChIP-seq |
| ☒ | ☐ Flow cytometry |
| ☒ | ☐ MRI-based neuroimaging |

# Antibodies

Antibodies used

The following commercial antibodies were used in this study:
1. anti-HA-HRP antibody (rat monoclonal, clone 3F10, Roche).
2. anti-FLAG-HRP antibody (mouse monoclonal, clone M2, Sigma Aldrich, F3165)

Validation

All used antibodies are commercially available and have been tested for specificity by the suppliers:

1. anti-HA-HRP antibody (rat monoclonal, clone 3F10, Roche): Anti-HA-Peroxidase, High Affinity (3F10) specifically recognizes the HA peptide sequence [YPYDVPDYA] derived from the influenza hemagglutinin protein. The antibody recognizes its antigenic determinant even when the HA peptide epitope is introduced into unrelated recombinant proteins by a technique known as epitope tagging.

2. anti-FLAG-HRP antibody (mouse monoclonal, clone M2, Sigma Aldrich, F3165): Monoclonal ANTI-FLAG® M2 is a purified immunoglobulin, IgG1, monoclonal antibody, isolated from a mouse cell culture, that binds to FLAG® fusion proteins.1 Unlike ANTI-FLAG® M1 antibody, the M2 antibody will recognize the FLAG® sequence at the N-terminus, Met-N-terminus, C-terminus, or at an internal site of FLAG® fusion proteins. Monoclonal ANTI-FLAG® M2 is useful for identification and capture of FLAG® fusion proteins by common immunological procedures such as Western blots and immuno-precipitation.

# Plants

Seed stocks

The plant lines used in this study were all previously generated. Information about the corresponding publications can be found in the Methods section. All plant lines used in this study are available upon request from the corresponding author.

Novel plant genotypes

*Describe the methods by which all novel plant genotypes were produced. This includes those generated by transgenic approaches, gene editing, chemical/radiation-based mutagenesis and hybridization. For transgenic lines, describe the transformation method, the number of independent lines analyzed and the generation upon which experiments were performed. For gene-edited lines, describe the editor used, the endogenous sequence targeted for editing, the targeting guide RNA sequence (if applicable) and how the editor was applied.*

Authentication

*Describe any authentication procedures for each seed stock used or novel genotype generated. Describe any experiments used to assess the effect of a mutation and, where applicable, how potential secondary effects (e.g. second site T-DNA insertions, mosiacism, off-target gene editing) were examined.*
