# Supplementary material for: Dual recognition of structurally unrelated mildew effectors underlies the broad-spectrum resistance of Pm3e in wheat
Source: Nat Commun. 2026 May 2;17:5955. doi: 10.1038/s41467-026-72199-w (PMC13342611; doi:10.1038/s41467-026-72199-w)
Supplement: Supplementary file 8 — Transparent Peer Review file [file 41467_2026_72199_MOESM8_ESM.pdf]

nature portfolio

Peer Review File

Dual recognition of structurally unrelated mildew effectors underlies the broad-spectrum resistance of Pm3e in wheat

Corresponding Author: Dr Marion Müller

This file contains all reviewer reports in order by version, followed by all author rebuttals in order by version.

Version 0:

Reviewer comments:

Reviewer #1

(Remarks to the Author)
In this manuscript the authors shows that the wheat Pm3d and Pme resistance gene alleles provide resistance to a broad set of diverse powdery mildew isolates and identify corresponding Avr loci recognised by these two receptors.
In the case of Pm33d, a corresponding Avr had already been identified, however it occurs in a tandem pair with the second duplicated copy carrying two amino acid changes and previously characterised as not recognised. Here the authors show that this variant is in fact weakly recognised in transient assays and corresponds to a genetic determinant of avirulence in the fungus itself.
In the case of Pm3e, which differs from Pm3d for only four amino acids, the authors make a remarkable finding that this receptor recognises two entirely different effectors. One of these is the same effector recognised by Pm3d, a typical member of the RALPH structural family which contains all of the so far characterised powdery mildew Avr proteins. However, the second Avr for Pm3e is an entirely unrelated protein that seems to define s different structural family of powdery mildew effectors. This finding of dual recognition of unrelated effectors by direct interaction (as opposed to a guard-type recognition) is highly significant and the first such example I am aware of.
Overall this is an excellent study, with the genetic analysis of pathogen strains complementing the single gene assays. I have no specific comments on the experiments or results presented, which are conducted to a very high standard and interpreted logically. While the text is by and large very well written, I find one aspect of the presentation somewhat misleading and serves to obscure and distract from the key finding. The text in several points equates the dual recognition by Pm3e of two unrelated effectors (truly novel and interesting) with the recognition by Pm3d of different versions of the same effector. I came away from the abstract thinking both receptors recognised entirely different effectors and that the 'combined specificity' receptor now recognised four different effectors. However, the two effectors in the reference genome that are recognised by Pm3d differ by only two amino acids, and there are several additional variants of these two genes that are also recognised and collectively they can all be linked by single amino acid changes. Thus, there is no true distinction between the 20069a and 20069b gene sequences. Rather than 'two effectors' recognised by Pm3d, there are either 6-8 different effectors (unique sequences differing by single aa's) or there is a single effector with multiple variants that can be recognised. This is a very similar situation to many other fungal avr genes, including others that the authors have worked on in powdery mildew. The presence of copy number variation at an Avr locus, in addition to allelic sequence divergence is also quite common in fungal pathogens. I suggest the authors remove all references to Pm3d recognising 'two effectors', as this creates confusion when the really significant result is that Pm3e truly does recognise two effectors that are completely unrelated. The abstract should also point out explicitly that one of the effectors recognised by Pm3e is the same as that recognised by Pm3d.

Reviewer #2

(Remarks to the Author)
This manuscript by Kunz et al. is a tour-de-force study of the resistance genes Pm3d and Pm3e, and the wider Pm3 family, from wheat. Alleles of Pm3, while showing a variety of resistance profiles, are largely defined as only having a small number of polymorphisms, located within the LRR region of the NLRs, and this suggests these regions as hot-spots for effector recognition. The Pm3e gene is shown to recognise structurally unrelated effector proteins from the powdery mildew fungus Blumeria graminis f. sp. tritici. This type of broad spectrum resistance is of high value to agriculture to break the boom-bust cycle of resistance protein deployment (or at least limit it). Further, Pm3d recognises 2 effectors from the RNase-like

structural family. Interestingly, the authors - through their mechanistic studies - are able to combine the Pm3d and Pm3e recognition profiles into a single gene, this highlights a potential for further engineering that could even further widen the use of these genes in resistance to powdery mildew in wheat. A major advantage of this work is the exploitation of transgenic wheat and fungal lines for robustness of the conclusions.

The authors begin their study by using existing transgenic wheat lines to clone the avirulence effectors recognised by Pm3e, with AvrPm3e_1 being a novel, large, effector with an unusual signal peptide region that implies this effector may be delivered to plant cells via an unconventional mechanism. The candidate effectors were validated in a standard N. benthamiama cell death assay which, while straight forward to perform, is remarkable in its consistency to reproduce the pairwise interactions seen in wheat challenged with the pathogen. The authors then investigated polymorphisms in the effector population via alphafold modelling and identified a surface region on the effector that is subject to changes. As an aside, the authors define a new family of Blumeria-specific effectors as AvrPm3e_1-like. A second RNase-like effector BgtE-20069b (previously characterised) was shown to be recognised by Pm3e in addition to Pm3d. Using similar approaches the authors show Pm3d also recognises two effectors, but they are both RNase-like.

As mentioned above, the authors complete their experiments to map the variation of Pm3 alleles to the LRR region and show that chimeric receptors can be generated that display both Pm3d and Pm3e specificities.

The authors include a comprehensive Methods section detailing the approaches used in this study.

Figs 2-7 are quite complex and take a little while to fully understand, but this is mainly due to the huge amount of work in this manuscript. Despite this comment, I do not think anything needs to be changed.

The Supplemental information is excellent and provides a critical resource to full understand the implications and impact of the work.

Overall, this is a well-presented manuscript that is exceptionally well written with clear and informative figures.

Version 1:

Reviewer comments:

Reviewer #1

(Remarks to the Author)
The authors have addressed my comments fully. I appreciate their clarifications and reasoning regarding the genetic versus molecular aspects of their descriptions.

Open Access This Peer Review File is licensed under a Creative Commons Attribution 4.0 International License, which permits use, sharing, adaptation, distribution and reproduction in any medium or format, as long as you give appropriate credit to the original author(s) and the source, provide a link to the Creative Commons license, and indicate if changes were made.
In cases where reviewers are anonymous, credit should be given to 'Anonymous Referee' and the source.
The images or other third party material in this Peer Review File are included in the article's Creative Commons license, unless indicated otherwise in a credit line to the material. If material is not included in the article's Creative Commons license and your intended use is not permitted by statutory regulation or exceeds the permitted use, you will need to obtain permission directly from the copyright holder.
To view a copy of this license, visit https://creativecommons.org/licenses/by/4.0/

Dear Editor,

Please find below our point-by-point response to the reviewer's comments on our manuscript entitled "Dual recognition of structurally unrelated mildew effectors underlies the broad-spectrum resistance of Pm3e in wheat."

In response to Reviewer #1, we have improved the manuscript by revising the text as suggested. Specifically, we have:

- Improved the wording related to the two AvrPm3d effectors to better reflect that they are highly similar, paralogous effectors.
- Revised the abstract to clarify that the Pm3e and Pm3d NLRs recognise a common AVR factor.

We are confident that these changes have significantly improved the clarity of the manuscript.

In addition, we also corrected a small mistake affecting Figure 3I. While compiling the Source Data file, we realised that we had accidentally used an outdated dataset to produce Figure 3I. The previous version of the figure therefore contained a slightly wrong size estimate for the two effector superfamilies. We corrected this error using the correct dataset and updated the reported size estimates for both effector superfamilies in Note S5 (Supplementary Material, L169/L170, 322 instead of 331 for AvrPm3e_1-like effectors and 130 instead of 150 for RALPH effectors). Importantly, the conclusion of this analysis remains unchanged, namely that AvrPm3e_1-like effectors are much larger in size than RALPH effectors.
We additionally corrected a mistake in the figure legend of Figure 3 in which the subfigures G-J were mentioned in the wrong order (L1052-L1058 of the revised manuscript).

Lastly, we added statements to the methods section and figure legends referencing source data and reporting of statistical analyses, to comply with the reporting policies of Nature Communications.

As there were no requests for additional experiments or analysis, there are no changes to the figures and no changes in the text in addition to the modifications described above.

Sincerely,

Lukas Kunz, Marion Müller, and Beat Keller

Reviewer #1 (Remarks to the Author):

In this manuscript the authors shows that the wheat Pm3d and Pme resistance gene alleles provide resistance to a broad set of diverse powdery mildew isolates and identify corresponding Avr loci recognised by these two receptors.
In the case of Pm33d, a corresponding Avr had already been identified, however it occurs in a tandem pair with the second duplicated copy carrying two amino acid changes and previously characterised as not recognised. Here the authors show that this variant is in fact weakly recognised in transient assays and corresponds to a genetic determinant of avirulence in the fungus itself.
In the case of Pm3e, which differs from Pm3d for only four amino acids, the authors make a remarkable finding that this receptor recognises two entirely different effectors. One of these is the same effector recognised by Pm3d, a typical member of the RALPH structural family

which contains all of the so far characterised powdery mildew Avr proteins. However, the second Avr for Pm3e is an entirely unrelated protein that seems to define s different structural family of powdery mildew effectors. This finding of dual recognition of unrelated effectors by direct interaction (as opposed to a guard-type recognition) is highly significant and the first such example I am aware of.
Overall this is an excellent study, with the genetic analysis of pathogen strains complementing the single gene assays. I have no specific comments on the experiments or results presented, which are conducted to a very high standard and interpreted logically. While the text is by and large very well written, I find one aspect of the presentation somewhat misleading and serves to obscure and distract from the key finding. The text in several points equates the dual recognition by Pm3e of two unrelated effectors (truly novel and interesting) with the recognition by Pm3d of different versions of the same effector. I came away from the abstract thinking both receptors recognised entirely different effectors and that the 'combined specificity' receptor now recognised four different effectors. However, the two effectors in the reference genome that are recognised by Pm3d differ by only two amino acids, and there are several additional variants of these two genes that are also recognised and collectively they can all be linked by single amino acid changes. Thus, there is no true distinction between the 20069a and 20069b gene sequences. Rather than 'two effectors' recognised by Pm3d, there are either 6-8 different effectors (unique sequences differing by single aa's) or there is a single effector with multiple variants that can be recognised. This is a very similar situation to many other fungal avr genes, including others that the authors have worked on in powdery mildew. The presence of copy number variation at an Avr locus, in addition to allelic sequence divergence is also quite common in fungal pathogens. I suggest the authors remove all references to Pm3d recognising 'two effectors', as this creates confusion when the really significant result is that Pm3e truly does recognise two effectors that are completely unrelated. The abstract should also point out explicitly that one of the effectors recognised by Pm3e is the same as that recognised by Pm3d.

We thank the reviewer for pointing out that our wording concerning the statement that Pm3d and Pm3e recognise two effectors could be misunderstood. We agree with the reviewer that, given the strong sequence similarity between the BgtE-20069a and BgtE-20069b effectors, they could be considered variants of the same effector, and we have indeed used such nomenclature in the past for effectors encoded by paralogous copies of other *AVR* genes in *Bgt*. However, in the present study, treating the two effector genes as a single entity and referring to their protein products as variants rather than as distinct AVR effectors throughout the manuscript would, in our opinion, not lead to the desired improvement in clarity of the text for the following two reasons:

1. The *Pm3* allele specificities towards the two avirulence genes are clearly distinct: *BgtE-20069b* is recognised by both *Pm3d* and *Pm3e* and thus genetically acts as *AvrPm3d* and *AvrPm3e*, whereas *BgtE-20069a* is exclusively recognised by *Pm3d* and therefore acts only as *AvrPm3d*. Therefore, while we agree with the reviewer that the distinction between the sequences of *BgtE-20069a* and *BgtE-20069b* effector genes is small, there is a clear genetic and functional difference between the two paralogous genes.
2. "What is the molecular basis of broad-spectrum (Pm3) resistance?" is one of the main research questions addressed in this manuscript. We show that both broad-spectrum resistance alleles *Pm3d* and *Pm3e* require adaptive changes in two *AVR* genes for resistance to be overcome by *Bgt*. For *AvrPm3d* genes in particular, we show that the molecular mechanisms resulting in functional loss of *AVRs* are clearly distinct between *BgtE-20069b* (deletion) and *BgtE-20069a* (amino acid polymorphism) and that only isolates combining both gain-of-virulence alleles can break *Pm3d* resistance.

Hence, in a genetic context, we consider it necessary to retain the notion in the manuscript that there are two *AvrPm3d* genes in *Bgt*. However, we agree that in several instances we did not sufficiently clarify that the two RALPH AVR effectors BgtE-20069a and BgtE-20069b recognized by Pm3d are in fact highly similar in sequence. Therefore, to enhance clarity and avoid misunderstandings, we have revised the statements indicating that Pm3d recognises “two effectors” and implemented additional textual changes throughout the manuscript as follows:

- We changed the wording in the abstract from “two AVR effectors” to “two *AVR* genes” (L30 of the revised manuscript) to reflect that, in this instance, we refer to the effector genes rather than the effector proteins. With this change, the statement is less likely to give the impression that the two entities represent distinct effector proteins and emphasizes that we are describing a genetic observation. Similarly, we revised the manuscript at positions L324-L329, L476-L478 and L483 to clarify that, in these instances, we refer to the *AVR genes* rather than to the proteins they encode.
- We clarified at several points throughout the manuscript, including the abstract, that the two *AvrPm3d* genes are paralogous and encode highly similar proteins (see L31, L101-L103, L300, L354-L356 and L1085/1086). Furthermore, in the context of NLR specificity, we now refer to them as BgtE-20069a/BgtE-20069b RALPH effector variants (L373-L375, L430, L541-L542) rather than as separate effector proteins to emphasize the high similarity between the BgtE-20069a and BgtE-20069b effectors.
- Lastly, as suggested by the reviewer, we revised the abstract to clarify that Pm3d and Pm3e recognise a common AVR (BgtE-20069b) (L29-L36), thereby avoiding the impression that Pm3d and Pm3e together recognise four distinct effectors.

Reviewer #2 (Remarks to the Author):

This manuscript by Kunz et al. is a tour-de-force study of the resistance genes Pm3d and Pm3e, and the wider Pm3 family, from wheat. Alleles of Pm3, while showing a variety of resistance profiles, are largely defined as only having a small number of polymorphisms, located within the LRR region of the NLRs, and this suggests these regions as hot-spots for effector recognition. The Pm3e gene is shown to recognise structurally unrelated effector proteins from the powdery mildew fungus Blumeria graminis f. sp. tritici. This type of broad spectrum resistance is of high value to agriculture to break the boom-bust cycle of resistance protein deployment (or at least limit it). Further, Pm3d recognises 2 effectors from the RNase-like structural family. Interestingly, the authors - through their mechanistic studies - are able to combine the Pm3d and Pm3e recognition profiles into a single gene, this highlights a potential for further engineering that could even further widen the use of these genes in resistance to powdery mildew in wheat. A major advantage of this work is the exploitation of transgenic wheat and fungal lines for robustness of the conclusions.

The authors begin their study by using existing transgenic wheat lines to clone the avirulence effectors recognised by Pm3e, with AvrPm3e_1 being a novel, large, effector with an unusual signal peptide region that implies this effector may be delivered to plant cells via an unconventional mechanism. The candidate effectors were validated in a standard N. benthamiama cell death assay which, while straight forward to perform, is remarkable in its consistency to reproduce the pairwise interactions seen in wheat challenged with the pathogen. The authors then investigated polymorphisms in the effector population via alphafold modelling and identified a surface region on the effector that is subject to changes. As an aside, the authors define a new family of Blumeria-specific effectors as AvrPm3e_1-

like. A second RNase-like effector BgtE-20069b (previously characterised) was shown to be recognised by Pm3e in addition to Pm3d. Using similar approaches the authors show Pm3d also recognises two effectors, but they are both RNase-like.

As mentioned above, the authors complete their experiments to map the variation of Pm3 alleles to the LRR region and show that chimeric receptors can be generated that display both Pm3d and Pm3e specificities.

The authors include a comprehensive Methods section detailing the approaches used in this study.

Figs 2-7 are quite complex and take a little while to fully understand, but this is mainly due to the huge amount of work in this manuscript. Despite this comment, I do not think anything needs to be changed.

The Supplemental information is excellent and provides a critical resource to full understand the implications and impact of the work.

Overall, this is a well-presented manuscript that is exceptionally well written with clear and informative figures.

We thank the reviewer for the positive comments.
